# Topiramate in the treatment of compulsive sexual behavior: case report

**DOI:** 10.1186/1471-244X-6-22

**Published:** 2006-05-23

**Authors:** Yasser Khazaal, Daniele Fabio Zullino

**Affiliations:** 1 Department of Psychiatry, Mood and Anxiety Disorders Unit, Echallens 9, 1004 Lausanne, Switzerland; 2 University Hospitals of Geneva, Division of Substance Abuse, Rue Verte 2, 1205 Geneva, Switzerland

## Abstract

**Background:**

Among the multiple mechanisms of action of topiramate, AMPA/kainate antagonism may be particularly interesting for the treatment of disorders characterized by conditioned cognitive and behavioral cue reactivity.

**Case presentation:**

We report the case of a patient consulting primarily for obesity and cue triggered snacking, who responded well on topiramate at doses up to 50 mg. Coincidentally he reported on an improvement of compulsive nonparaphilic sexual behaviors (consumption of prostitution), which was also strongly triggered by environmental cues. Both addictive behaviors (snacking and consumption of prostitution) reoccurred after discontinuation of topiramate and again responded reintroduction of the drug.

**Conclusion:**

The present case report of topiramate's effect on comorbid obesity and nonparaphilic addiction could be interpreted as a further indication that topiramate acts on the common pathway underlying conditioned behaviors and seems to be a treatment of behavioral disorders associated with environmental cues.

## Background

Behaviors with a compulsive and/or addictive component can characteristically be triggered by environmental cues [1]. Recent research has highlighted a central role of the glutamatergic system in mediating this cue reactivity.

The α-amino-3-hydroxy-5-methyl-4-isoxazolepropionic acid (AMPA) receptor has been found to mediate the expression of established addiction [2]. For example., AMPA has been found to reinstate drug seeking [3] and AMPA receptor blocking properties (AMPAR) antagonists to block reinstatement[4].

The sulfamate derivative, topiramate, has multiple mechanisms of action, including AMPA/kainate blocking properties [5]. Given the role of AMPAR in the expression of conditioned responses and topiramate's AMPAR-antagonistic properties, topiramate's interest in the treatment of disorders characterized by conditioned cognitive and/or behavioral responses is suggested [6].

Diverse studies and case reports have recently raised the interest of topiramate in the treatment of addictive disorders such as alcohol dependence [7,8], opiates [9] and benzodiazepines withdrawal [10], smoking cessation (Khazaal et al in press), but also in other disorders characterized by compulsive behavior like binge eating disorder [11], bulimia nervosa [12], obesity [13], Tourette's syndrome [14], flash-backs and nightmares in PTSD [15], and self-mutilation behavior [16].

Nonparaphilic sexual addiction has been characterized by excessive and continued engagement in conventional sexual activities (i.e extra marital affairs, prostitution, pornography, compulsive masturbation) despite subjective distress and negative consequences [17]. Traditional treatment approaches include individual or group CBT and family therapy. To date, there is no well studied pharmacological treatment of nonparaphilic sexual addictions. Some reports indicate the utility of lithium, tricyclic antidepressants, selective serotonine reuptake inhibitors, nefazodone and atypical antipsychotics and fluoxetine-naltrexone combination [18-21].

To our knowledge, the effectiveness of topiramate is reported in a paraphilic[22] as well as in a nonparaphilic sexual disorder [23]. We are presenting what is believed to be the first report of topiramate effect on comorbid obesity and nonparaphilic sexual addiction.

The authors obtained written consent from the patient for publication of this case report.

## Case presentation

The patient, a 33 years old, Caucasian, non-smoker, reported no personal history of substance abuse, gambling or mood or anxiety disorders. He had been married for two years, did not have any children and worked as an engineer for the same company for 4 years. He reported a good quality of marital life and satisfying sexual marital activities.

He currently consulted for the treatment of obesity (Body mass index: BMI = 31). He reported three previous dietary treatments and one cognitive behavioral treatment, which were unfortunately always immediately followed by weight gain. He asked for pharmacological treatment of his obesity.

The assessment of his eating behavior revealed no binge eating. While he took 3 meals per day, the frequent snacks (5 to 7 per day) represented half of his daily caloric intake. The intake of snacks was always triggered by specific cues (such as specific food stimuli: sweets with vanilla cream or chocolate and sandwiches with some kind of bread, the smell of hot bread, some roads, specific food stores...).

The patient provided verbal informed consent based on disclosure of off-label usage of topiramate for the indication, the alternative treatments, and expected adverse events and risks. Topiramate was started at a dosage of 25 mg and then increased after one week to a dosage of 50 mg. He also received three cognitive and behavioral sessions in order to explore specific cues and to determine their role in his obesity. He was advised to maintain his mean meals, and snacking reduction and weight loss were defined as treatment goals.

After one week of topiramate treatment, he has succeeded in reducing "snacking" to twice a week. He reported that although he saw the cues he felt less attracted by them, sometimes even failing to notice them. He did not experience significant side effects and lost 3.5 kg during the first month.

What's more, the patient reported an unexpected change in his sexual behavior. He described for the first time during the second month of treatment that he began going to massage clubs, and street prostitutes 4 years ago which led him quickly to spend nearly one third of his monthly income : 3000 Swiss Francs (F). He did not engage in pornography nor paraphilic behaviors such as exhibitionism, voyeurism or pedophilia. Furthermore he did not have any extra marital sexual relations other than through prostitution. He tried many times to stop or reduce this behavior due to shame and feelings of guilt and in order to reduce his expenses but he could not stop this behavior for more than one week. The subject reported that seeing sexual advertisements or passing through streets where prostitutes work caused in him a feeling of urgency, craving with an inner tension accompanied by a hot abdominal sensation and modification in salivation lasting until his next sexual intercourse with a prostitute. He felt an attraction to those places, "always returning there as if drawn by a magnet". Surprisingly, he observed in the second week of topiramate treatment that he was in an attractive area (with many street prostitutes) but did not feel any urge. He experienced this phenomenon later when trying to expose himself to habitual cuesnoticing the places and the signs but feeling less attracted by them and no longer having the craving nor physical symptoms, urges and tension. His marital sexual activity remained unchanged and was perceived as good. He spent no more than 200 F per month in massage clubs and was satisfied by this evolution.

After 4 months of treatment, a weight loss of 10.8 kg (BMI = 27.3) and persistent modification in the sexual addiction, he decided to stop treatment, feeling that he had more than reached his goals.

Eight weeks after topiramate discontinuation, he consulted again describing a relapse in the compulsive eating and compulsive prostitute visits, having occurred two weeks after topiramate cessation, leading to a weight gain of 3 kg and spending of 2000 F in brothels. Three weeks after this relapse, he restarted topiramate on his own, and stated a reinstatement of the therapeutic effect within 10 days. He asked for a new topiramate prescription and remained on the same treatment (topiramate 50 mg/day) for the following 6 months. His weight remained stable (BMI = 26.7) and he spent less than 300 F/month for prostitutes with no relapse to the previous feeling of irrepressible attraction of related sexual cues.

## Conclusion

For the two problematic behaviors, he reported on a reduced urge and craving and attraction of environmental cues after topiramate introduction which allowed him to reduce considerably these behaviors.

Previous studies using topiramate for treating addictive behaviors used doses reached 200 mg [7,24]. The prefixed schedules used by those studies, however, did not allow the adapted doses to be determined. In our case, we found a dose as low as 50 mg to be sufficient, in contrast to the previous report of topiramate's effect on sexual non paraphilic addiction, where 200 mg per day were used.

While a spontaneous remission or a placebo effect cannot be completely ruled out in the present case, different criteria sustain topiramate efficacy: the previous case reports, the temporal correlation with the topiramate administration, the relapse after discontinuation and the responding after resumption of the treatment, and the absence of any contributing life event.

The concomitant improvement of eating habits and sexual addiction observed under topiramate corroborate the hypothesis of topiramate acting on the common pathway underlying conditioned behaviors. As expected by psychopharmacological data and previous clinical trials on the treatment of addictive and eating disorders, topiramate seems to be promising medication for treatment of sexual addiction associated with behavioral cues.

## Competing interests

The author(s) declare that they have no competing interests.

## Authors' contributions

YK has made important contributions to the case observation, the conception of the manuscript and its redaction.

DFZ has been involved in drafting the manuscript and has revised the main intellectual content.

All authors have read and approved the final manuscript.

## Pre-publication history

The pre-publication history for this paper can be accessed here:



## References

[B1] Balfour ME, Yu L, Coolen LM (2004). Sexual behavior and sex-associated environmental cues activate the mesolimbic system in male rats. Neuropsychopharmacology.

[B2] Jackson A, Mead AN, Stephens DN (2000). Behavioural effects of alpha-amino-3-hydroxy-5-methyl-4-isoxazolepropionate-receptor antagonists and their relevance to substance abuse. Pharmacol Ther.

[B3] Cornish JL, Duffy P, Kalivas PW (1999). A role for nucleus accumbens glutamate transmission in the relapse to cocaine-seeking behavior. Neuroscience.

[B4] Vorel SR, Liu X, Hayes RJ, Spector JA, Gardner EL (2001). Relapse to cocaine-seeking after hippocampal theta burst stimulation. Science.

[B5] Gibbs JWIII, Sombati S, DeLorenzo RJ, Coulter DA (2000). Cellular actions of topiramate: blockade of kainate-evoked inward currents in cultured hippocampal neurons. Epilepsia.

[B6] Zullino DF, Krenz S, Besson J (2003). AMPA blockade may be the mechanism underlying the efficacy of topiramate in PTSD. J Clin Psychiatry.

[B7] Johnson BA, Ait-Daoud N, Bowden CL, DiClemente CC, Roache JD, Lawson K, Javors MA, Ma JZ (2003). Oral topiramate for treatment of alcohol dependence: a randomised controlled trial. Lancet.

[B8] Johnson BA (2004). Topiramate-induced neuromodulation of cortico-mesolimbic dopamine function: a new vista for the treatment of comorbid alcohol and nicotine dependence?. Addict Behav.

[B9] Zullino DF, Krenz S, Zimmerman G, Miozzari A, Rajeswaran R, Kolly S, Khazaal Y (2005). Topiramate in opiate withdrawal- comparison with clonidine and with carbamazepine/mianserin. Subst Abus.

[B10] Cheseaux M, Monnat M, Zullino DF (2003). Topiramate in benzodiazepine withdrawal. Hum Psychopharmacol.

[B11] McElroy SL, Arnold LM, Shapira NA, Keck PEJ, Rosenthal NR, Karim MR, Kamin M, Hudson JI (2003). Topiramate in the treatment of binge eating disorder associated with obesity: a randomized, placebo-controlled trial. Am J Psychiatry.

[B12] Hoopes SP, Reimherr FW, Hedges DW, Rosenthal NR, Kamin M, Karim R, Capece JA, Karvois D (2003). Treatment of bulimia nervosa with topiramate in a randomized, double-blind, placebo-controlled trial, part 1: improvement in binge and purge measures. J Clin Psychiatry.

[B13] Bray GA, Hollander P, Klein S, Kushner R, Levy B, Fitchet M, Perry BH (2003). A 6-month randomized, placebo-controlled, dose-ranging trial of topiramate for weight loss in obesity. Obes Res.

[B14] Abuzzahab FS, Brown VL (2001). Control of Tourette's syndrome with topiramate. Am J Psychiatry.

[B15] Berlant JL (2004). Prospective open-label study of add-on and monotherapy topiramate in civilians with chronic nonhallucinatory posttraumatic stress disorder. BMC Psychiatry.

[B16] Cassano P, Lattanzi L, Pini S, Dell'Osso L, Battistini G, Cassano GB (2001). Topiramate for self-mutilation in a patient with borderline personality disorder. Bipolar Disord.

[B17] Goodman A (1993). Diagnosis and treatment of sexual addiction. J Sex Marital Ther.

[B18] Coleman E, Gratzer T, Nesvacil L, Raymond NC (2000). Nefazodone and the treatment of nonparaphilic compulsive sexual behavior: a retrospective study. J Clin Psychiatry.

[B19] Kafka MP (1994). Sertraline pharmacotherapy for paraphilias and paraphilia-related disorders: an open trial. Ann Clin Psychiatry.

[B20] Kafka MP, Prentky R (1992). Fluoxetine treatment of nonparaphilic sexual addictions and paraphilias in men. J Clin Psychiatry.

[B21] Raymond NC, Grant JE, Kim SW, Coleman E (2002). Treatment of compulsive sexual behaviour with naltrexone and serotonin reuptake inhibitors: two case studies. Int Clin Psychopharmacol.

[B22] Shiah IS, Chao CY, Mao WC, Chuang YJ (2006). Treatment of paraphilic sexual disorder: the use of topiramate in fetishism. Int Clin Psychopharmacol.

[B23] Fong TW, De La GR, Newton TF (2005). A case report of topiramate in the treatment of nonparaphilic sexual addiction. J Clin Psychopharmacol.

[B24] Kampman KM, Pettinati H, Lynch KG, Dackis C, Sparkman T, Weigley C, O'Brien CP (2004). A pilot trial of topiramate for the treatment of cocaine dependence. Drug Alcohol Depend.

